# Natural sugar feeding rates of *Anopheles* mosquitoes collected by different methods in western Kenya

**DOI:** 10.1038/s41598-022-25004-9

**Published:** 2022-11-29

**Authors:** Seline Omondi, Jackline Kosgei, Silas Agumba, Brian Polo, Nick Yalla, Vincent Moshi, Bernard Abong’o, Maurice Ombok, Daniel P. McDermott, Julian Entwistle, Aaron M. Samuels, Feiko O. Ter Kuile, John E. Gimnig, Eric Ochomo

**Affiliations:** 1 Entomology Section, Centre for Global Health Research, Kenya Medical Research Institute, P.O. Box 1578-40100, Kisumu, Kenya; 2 Vector Group, Liverpool School of Tropical Medicine, Liverpool, UK; 3 Consultant to Innovative Vector Control Consortium, Liverpool, UK; 4 Centers for Disease Control and Prevention, Kisumu, Kenya; 5 Division of Parasitic Diseases and Malaria, Centers for Disease Control and Prevention, Atlanta, GA USA; 6 Department of Clinical Sciences, Liverpool School of Tropical Medicine, Liverpool, UK

**Keywords:** Entomology, Malaria

## Abstract

Attractive targeted sugar baits (ATSBs) are a potential vector control tool that exploits the sugar-feeding behaviour of mosquitoes. We evaluated the sugar-feeding behaviour of *Anopheles* mosquitoes as part of baseline studies for cluster randomised controlled trials of ATSBs. Mosquitoes were collected indoors and outdoors from two villages in western Kenya using prokopack aspirations, malaise tent traps and ultraviolet (UV) light traps. Individual mosquitoes were subjected to the cold anthrone test to assess the presence of sugar. Overall, 15.7% of collected mosquitoes had fed on natural sugar sources. By species and sex, the proportion sugar-fed was 41.3% and 27.7% in male and female *Anopheles funestus,* 27.2% and 12.8% in male and female *An. arabiensis,* and 9.7% and 8.3% in male and female *An. coustani,* respectively**.** Sugar-feeding was higher in unfed than blood-fed mosquitoes and higher in male than gravid mosquitoes. *Anopheles* mosquitoes obtained sugar meals from natural sources during all physiological stages, whether they rest indoors or outdoors. These findings offer a potential avenue to exploit for the control of mosquitoes, particularly with the advent of ATSBs, which have been shown to reduce mosquito densities in other regions.

## Introduction

The scale-up of malaria control since 2000 has resulted in substantial reductions in the burden of malaria globally. Modelling studies suggest that much of the reduction was due to vector control efforts, particularly long-lasting insecticidal nets (LLINs) and indoor residual spraying (IRS)^1^. However, despite these gains, malaria remains endemic in sub-Saharan Africa and is one of the leading causes of morbidity and mortality^2^. Furthermore, recent reports indicate that the decline in malaria burden has stalled or reversed in some areas^3^. There is a need for additional control tools to be integrated with the existing approaches^2,4^.

Attractive targeted sugar baits (ATSBs) are a potential new vector control tool that exploits the sugar feeding behaviour of mosquitoes. While female mosquitoes require blood for egg maturation, both males and females require sugar to meet energy needs. Mosquitoes obtain sugar from various sources, including floral and extra-floral nectaries, fruits and honeydew^5–8^. In West Africa, it has been demonstrated that sugar baits comprised of juice from local fruits are attractive to mosquitoes^9,10^ which may be exploited to target mosquitoes responsible for transmitting vector-borne diseases in humans or domestic animals. The efficacy of sugar baits spiked with an oral toxin was first demonstrated in field trials in Israel against *An. claviger* in underground cisterns^11^ and against *An. sergentii* and *An. caspius* in a desert oasis^12^. Subsequently, a small-scale trial in Mali where vegetation was sprayed with a sugar solution plus boric acid resulted in an estimated 90% reduction in older mosquitoes^13^. More recently, a larger trial using a prototype of a commercial bait station also resulted in substantial reductions^14^, suggesting that ATSBs could be a promising new malaria control tool.

However, ATSBs have only been evaluated in areas characterised by an arid climate with limited vegetation, where ATSBs may face little competition from natural sugar sources. In more tropical climates, mosquitoes are thought to have access to a wider variety of natural sugar sources, which may limit feeding on ATSBs and/or require alternative placement strategies. To better understand the potential for ATSBs in areas with a wider variety of natural sugar sources, we characterised the sugar feeding behaviour of the primary *Anopheles* vectors of malaria as part of baseline studies for population-based controlled trials of ATSBs in western Kenya where malaria transmission is high and perennial^15^.

## Methodology

### Study sites

This study was conducted in Mabinju (-0.1849075, 34.3727141) and Abidha (-0.1552102, 34.4014668) villages in Rarieda sub-County, Siaya County, western Kenya. The area is part of the KEMRI-CDC Health and Demographic Surveillance System (HDSS)^16^ where a population of 262,215 as of 2019 has been continuously monitored for in- and out-migration and births and deaths and where regular surveys of malaria prevalence have been conducted for over a decade^17^. Mabinju and Abidha were selected as historically, high numbers of *Anopheles* mosquitoes have been observed in these villages^18,19^.

Most people in the study area are of the Luo ethnic group and earn their living through subsistence farming and fishing. The primary vectors in this area are *An. arabiensis*, *An*. *gambiae* sensu stricto and *An*. *funestus*. Malaria transmission is highest following the long rains which occur from April to June with a smaller peak following the short rains in October/November. However, rainfall and malaria transmission may occur throughout the year. The study area was the site of a large-scale trial of insecticide-treated nets (ITNs) and now receives LLINs through periodic mass campaigns and routine distribution to pregnant women and children through antenatal clinics and child welfare clinics^18,20–22^. In the 2020 Malaria Indicator Survey, 77.9% of households in the Lake-Endemic region of western Kenya owned at least one net^23^. In a study in this area in 2015, LLIN usage across all ages was 87.0% and 91.2% in children under 5 years. Despite high coverage of LLINs malaria prevalence by microscopy was 36.1% across all ages and 39.0% among children under 5 years^15^.

### Mosquito sampling

Mosquito sampling was done in and around 10 randomly selected dwellings (structures) in each village for 2 weeks each month during the cool dry season between July and September 2020 using three trapping methods. Ultraviolet (UV) light traps (Model 512, John W. Hock Company, Gainesville, Florida, USA) were used to collect mosquitoes in three positions in or outdoors near each selected dwelling (structure): (1) Indoors (B), (2) 10 m outdoors from the structure (outdoors near to structure; UVLT-C) and (3) immediately outside the compound (outdoors-outside compound; UVLT-D) at a distance of 15 to 20 m from the structure with an indoor trap. Indoors, the traps were set 1.5 m above the ground at the foot of an occupied bednet. Indoor traps were set at 17:00 and collected at 07:00 the following morning to avoid inconveniencing the household members**.**

Outdoor traps were suspended from unoccupied buildings and trees approximately 1.5 m above the ground and sheltered from the rain. The traps were placed away from shelters where animals were kept and no attractant other than the UV lights were deployed with the traps. The collection cups from these outdoor light traps were collected hourly from 17:00 to 07:00. Mosquitoes were also collected using a single standard 6 m malaise tent trap (Model 3012, John W. Hock Company, Gainesville, Florida, USA) set approximately 50 m from the nearest structure in an open field within Mabinju village. The malaise trap collections were done in tandem with the outdoor UV light traps. Resting collections were done in the mornings from 07:00 to 11:00 using prokopack (Model 1419, John W. Hock Company, Gainsville, Florida, USA) aspirators indoors and outdoors. Outdoor aspirations were done in and around clay pots and other water storage containers outside the structure. Collections were done by moving the prokopack aspirators under the eaves and within dark corners around the structure and vegetation where mosquitoes were likely to rest. Mosquito collections indoors were done to determine the natural sugar-feeding rate of host-seeking and resting mosquitoes for UVLT and prokopack aspiration respectively.

Collections done outdoors using UVLT near and outside the compound were to determine the sugar-feeding rate of mosquitoes closer to the dwelling. Malaise tent trap collections were to determine the sugar-feeding rate of mosquitoes free flying in the wild.

### Mosquito processing

For processing, mosquitoes were transported to the field laboratory in Rarieda sub-County. Transportation from the collection sites to the field laboratory took approximately 30 min and lab processing took another 30 min. Upon arrival at the lab, live mosquitoes were knocked down either in chloroform or by freezing at − 20 °C. *Anopheles* mosquitoes were identified to species level by trained technician using dissecting microscopes and *Anopheles* taxonomic keys^24^; the sex and abdominal status of females (blood fed, non-blood fed, or gravid) were also recorded. The alcohol precipitation method and conventional polymerase chain reaction were used to speciate a subset of *Anopheles gambiae* sl to sibling species^25,26^.

Individual *Anopheles* mosquitoes were subjected to the cold anthrone test^27^ to assess the presence of sugar. The anthrone reagent was prepared as follows: 500 ml of 72% sulphuric acid was made by adding acid to distilled water, after which 1 g of anthrone powder was added to the solution. The mixture turned to a yellow solution after mixing. Individual mosquitoes were placed in a 96-well round-bottomed ELISA plate. The mosquitoes were then crushed singly using pestles, and 200 µl of the cold anthrone reagent was added. The plates were incubated for 45 min at room temperature, after which the results were scored based on the intensity of the green–blue colouration: yellow (no change in colour), light green, green and dark green. Yellow was interpreted as mosquitoes that were not sugar-fed, while light green, green and dark green indicated increasing levels of sugar feeding (scored as 0, 1, 2 and 3).

All *Anopheles* mosquitoes were included in anthrone testing independent of their abdominal status and sex. However, only *An. gambiae*, *An. funestus* and *An. coustani* were included for further analysis as inadequate numbers of the other species were available. For all analyses, gravid and half-gravid mosquitoes were combined into a single category referred to hereafter as “gravid”.

### Data analysis

Data was recorded in a Microsoft Excel spreadsheet with the date, collection method, location, collection hour, species, sex, abdominal status and anthrone result. The anthrone result was also converted to a binary outcome (any level of sugar feeding versus no sugar feeding). Logistic regression was used to compare feeding rates between species, collection method, abdominal status, and time of collection. All models were adjusted for repeated measures on mosquitoes from the same collection (location, structure, and date) using generalised estimating equations (GEE). All statistical analyses were carried out using SAS version 9.4 and R statistical package version 4.0.3.

### Ethical considerations

The study was reviewed and approved by the Kenya Medical Research Institute Scientific and Ethics Review Unit (SERU 3613) and by the Institutional Review Board of the Liverpool School of Tropical Medicine Research Ethics Committee (18-015). The study was also approved through a reliance agreement between the IRB of the US Centers for Disease Control and Prevention and KEMRI SERU (CDC IRB 7112). Permission to conduct the study was also obtained from the respective leaders and elders of each of the two villages. Informed consent was obtained verbally from inhabitants of the structures where mosquito collections were conducted in English, Kiswahili or Dholuo.

## Results

### *Anopheles* species variation

A total of 18,386 *Anopheles* mosquitoes were collected from the two villages over the 3-month collection period, with 2 weeks of sampling each month. Eight different *Anopheles* species were identified though most were *An. coustani* (n = 11,623; 63%), *An. funestus* (n = 5049; 27%) or *An. gambiae* s.l. (n = 1572; 9%)*.* By PCR, most *An. gambiae* s.l. were *An. arabiensis* (n = 275; 96%), the rest being *An. gambiae* s.s. Other species of *Anopheles* mosquitoes that were collected only outdoors included *An. maculipalpis* (N = 4), *An. pharoensis* (N = 114), *An. rufipes* (N = 10), *An. squamosus* (N = 11) and *An. parensis* (N = 3). The total number of mosquitoes tested by species, abdominal status and collection method/location are presented in Table 1.

**Table 1:** The number of *An. gambiae*, *An. funestus* and *An. coustani* collected using different trapping methods and tested for sugar by cold anthrone test.

Species	Collection method	Male	Non-blood fed	Blood fed	Gravid*
*An. funestus*	Aspiration indoor	1039	342	452	460
	Aspiration outdoor	122	60	24	17
	Malaise	11	12	0	0
	UVLT indoor	615	1258	19	58
	UVLT outdoor (C)	30	279	5	5
	UVLT outdoor (D)	39	193	2	7
*An. gambiae*	Aspiration indoor	34	18	34	28
	Aspiration outdoor	28	22	19	4
	Malaise	8	22	0	0
	UVLT indoor	61	191	16	8
	UVLT outdoor (C)	64	537	5	8
	UVLT outdoor (D)	59	393	1	12
*An. coustani*	Aspiration indoor	0	5	0	0
	Aspiration outdoor	2	11	6	1
	Malaise	10	101	6	3
	UVLT indoor	28	464	75	2
	UVLT outdoor (C)	489	3716	451	25
	UVLT outdoor (D)	958	4895	359	16

*Includes both gravid and half gravid.

### Sugar feeding

#### The proportion and intensity of sugar feeding by species

Overall, 15.7% of all the mosquitoes caught using the different trapping methods had fed on natural sugar sources. *Anopheles funestus* males and females had the highest rate of sugar feeding with 41.3% and 27.7%, respectively. For *An. gambiae* s.l.*,* 27.2% of males and 12.8% of females had fed on sugar, while this was 9.7% and 8.3% for male and female *An. coustani***.** These interspecific trends were similar when sugar feeding was classified based on the intensity of the anthrone result **(**Table 2**)**. Sugar feeding rates were significantly higher in *An. funestus* compared to both *An. gambiae* s.l. and *An. coustani* for both males and females while sugar feeding rates in *An. gambiae* s.l. were significantly higher than those of *An. coustani* (*p* < 0.05 for all comparisons; Supplementary Tables 1a & 1b).

**Table 2:** The intensity of sugar feeding by species. Zero is interpreted as unfed mosquitoes while increasing intensities of sugar feeding are indicated as 1, 2 and 3.

Species	Anthrone result	Males		Females	
		Number	Percent (95% CI)	Number	Percent (95% CI)
*An. funestus*	0	1090	58.7 (51.4–66)	2310	72.3 (69.7–75)
	1	364	19.6 (15.5–23.7)	380	11.9 (10.4–13.4)
	2	234	12.6 (10.1–15.1)	243	7.6 (6.5–8.7)
	3	168	9.1 (6.9–11.3)	260	8.1 (6.8–9.5)
	Any sugar	766	41.3 (34.0–48.6)	883	27.7 (25.0–30.3)
*An. gambiae*	0	185	72.8 (64.3–81.3)	1149	87.2 (84.3–90)
	1	23	9.1 (5.1–13)	105	8.0 (5.5–10.4)
	2	20	7.9 (4.1–11.7)	32	2.4 (1.6–3.2)
	3	26	10.2 (5.4–15.1)	32	2.4 (1.5–3.4)
	Any sugar	69	27.2 (18.7–35.7)	169	12.8 (10.0–15.7)
*An. coustani*	0	1343	90.3 (88.5–92.1)	9296	91.7 (90.8–92.6)
	1	88	5.9 (4.7–7.2)	609	6.0 (5.3–6.7)
	2	27	1.8 (1.1–2.6)	171	1.7 (1.4–2)
	3	29	2.0 (1.2–2.7)	60	0.6 (0.4–0.7)
	Any sugar	144	9.7 (7.9–11.5)	840	8.3 (7.4–9.2)

#### Sugar feeding by sex and abdominal status

Higher rates of sugar feeding were observed in males compared to females and in non-blood fed females compared to blood-fed mosquitoes across all the species. Sugar feeding rates among gravid females varied by species, with rates in *An. funestus* similar to those of blood-fed females and rates in *An. coustani* were similar to non-blood fed mosquitoes. Sugar feeding rates among gravid *An. gambiae* s.l. were intermediate between blood-fed and non-blood fed females (Table 3). In *An. funestus,* higher sugar feeding rates were observed in males and non-blood fed females compared to blood-fed and gravid mosquitoes (*p* < 0.001 for all comparisons). Sugar feeding rates were not significantly different between *An. funestus* males and non-blood fed females (*p* = 0.105) or between blood-fed and gravid females (*p* = 0.596) (Supplementary Tables 2a & 2b). Sugar feeding among male *An. gambiae* was significantly higher than blood-fed, non-blood fed or gravid females of the same species (*p* < 0.01 for all comparisons). Non-blood fed *An. gambiae* s.l. were significantly more likely to have sugar compared to blood-fed *An. gambiae* s.l. (*p* = 0.038). There was no significant difference in sugar feeding rates between gravid *An. gambiae* s.l. and either blood-fed (*p* = 0.184) or non-blood fed *An. gambiae* s.l. (*p* = 0.523) (Supplementary Tables 3a & 3b). Sugar-feeding rates were higher among male than female *Anopheles coustani* (*p* < 0.001), and higher among non-blood-fed females than blood-fed females (*p* < 0.001). There were no other statistically significant pairwise comparisons for *An. coustani* (Supplementary Tables 4a & 4b).

**Table 3:** Frequency of sugar feeding by species, sex and abdominal status.

Status	*An. gambiae*		*An. funestus*		*An. coustani*	
	Number	Percent (95% CI)	Number	Percent`(95% CI)	Number	Percent (95% CI)
Nonblood-fed	1183	13.4 (10.4–16.5)	2144	30.4 (27.2–33.5)	9192	8.6 (7.6–9.6)
Blood-Fed	75	5.3 (0.6–10.1)	502	21.9 (17.9–26)	897	4.9 (3.3–6.5)
Gravid	60	10 (2.8–17.2)	547	22.3 (17.6–27)	47	8.5 (0.9–16.1)
Male	254	27.2 (18.7–35.7)	1856	41.3 (34.0–48.6)	1487	9.7 (7.9–11.5)

#### Sugar feeding rates by collection method and location

**Figure 1:**
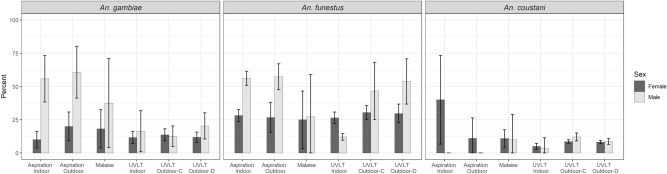
Sugar feeding rates of *An. gambiae*, *An. funestus* and *An. coustani* by collection method, location, species, and sex. Error bars represent the 95% confidence limits.

Sugar feeding rates by species, sex and collection method are provided in Fig. 1. Sugar feeding rates among female *An. funestus* or female *An. gambiae* s.l. were not significantly different based on method and/or collection location (*P* > 0.05 for all comparisons; Supplementary Tables 5a & 5b and 7a &7b). For male *An. funestus*, sugar feeding rates were highest among those collected by aspiration, indoors or outdoors, and UVLT, both close to and more distant from the structure. The lowest sugar feeding rates were observed among mosquitoes collected by UVLT indoors. In pairwise comparisons, sugar feeding rates were significantly lower among male *An. funestus* collected by UVLT indoors compared to those collected by aspiration indoors, aspiration outdoors, UVLT-C (close to the structure) or UVLT-D (distant from structure) (*p* < 0.001 for all comparisons; Supplementary Tables 6a & 6b). Male *An. gambiae* s.l. collected by aspiration indoors or outdoors were significantly more likely to be sugar-fed compared to those captured by UVLT indoors (*p* < 0.05 for both comparisons), UVLT-C (*p* < 0.001 for both comparisons), and UVLT-D (*p* < 0.003 for both comparisons). No other pairwise comparisons of sugar feeding by collection method were statistically significant among *An. gambiae* males.

For *An. coustani* females, the highest rates of sugar feeding were observed among those collected by indoor aspiration (40.0%), although only 5 mosquitoes were available for testing. Despite the low numbers, sugar feeding rates were significantly higher in *An. coustani* collected by indoor aspiration compared to malaise traps, UVLT indoor, UVLT-C, and UVLT-D (*p* < 0.02 for all comparisons; Supplementary Tables 9a & 9b). Low numbers of *An. coustani* males were available for testing, and only six pairwise comparisons were possible. None were statistically significant.

#### Sugar feeding by time of collection

**Figure 2:**
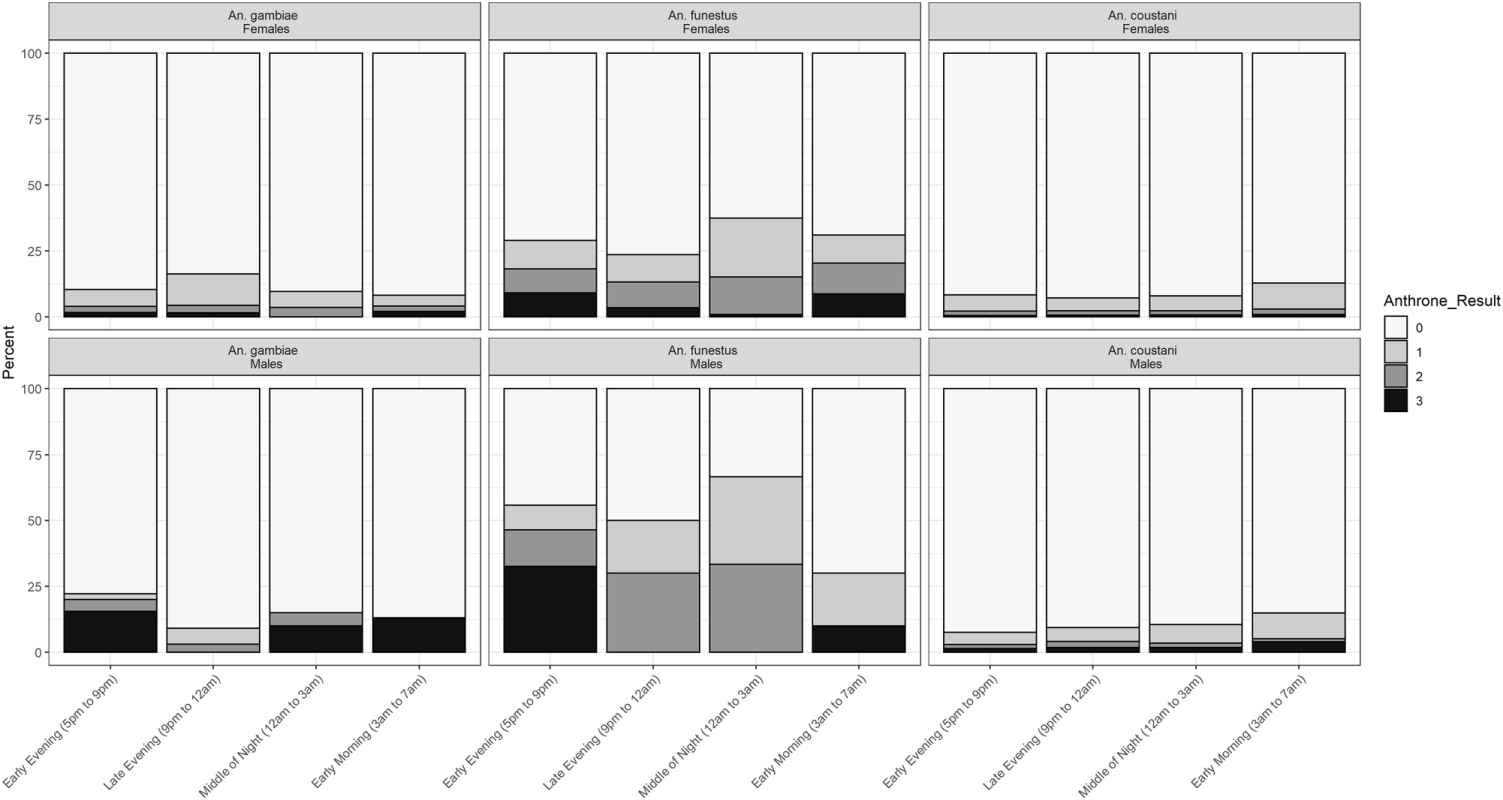
Sugar feeding intensity by the time. Anthrone results (0 = no sugar, 1, 2 and 3 = increasing levels of sugar feeding).

Due to high variation in mosquito numbers from 1 h to the next, outdoor hourly data were grouped into categories of 3 or 4 h: early evening (5–9 pm), late evening (9 pm to 12 am), middle of the night (12–3 am), or early morning (3–7 am) (Fig. 2). For *An. funestus* and *An. gambiae* s.l., there was little evidence of differences in the proportion of sugar-fed mosquitoes by time. The only statistically significant pairwise comparison was for *An. funestus* females collected in the middle of the night, (12–3 am) which were significantly more likely to be sugar-fed than *An. funestus* females collected in the late evening (9 pm to 12 am) (*p* = 0.022). No other pairwise comparisons for *An. funestus* or *An. gambiae* were statistically significant (Supplementary tables 11a, 11b, 12a, 12b, 13a, 13b, 14a & 14b).

Female *An. coustani* collected between 3 and 7 am were significantly more likely to be sugar-fed than those collected in the early evening (5–9 pm), late evening (9 pm to 12 am), and the middle of the night (12–3 am) (*P* < 0.01 for all comparisons; Supplementary Tables 15a & 15b). For *An. coustani* males, the sugar-fed proportion was also highest in the early morning. The differences were statistically significant when compared to feeding rates among *An. coustani* males collected in the early evening (5–9 pm; *p* = 0.010) or in the late evening (9 pm to 12am; *p* = 0.036) (Supplementary Tables 16a & 16b).

## Discussion

This study assessed the natural sugar feeding behaviour of *Anopheles* malaria vectors in western Kenya to identify potential aspects that could be exploited in the design and deployment of ATSBs, and the potential for natural sugar sources to compete against ATSBs. Sugar feeding was observed across all the main malaria vectors, in both males and females in different physiological states. Furthermore, sugar-fed mosquitoes were detected by several different trapping methods deployed indoors and outdoors. However, there was variation in sugar feeding among the three species tested, between the males and females, and between females in different physiological states. There were also differences in the proportion of sugar-fed mosquitoes by trapping methods, though this varied by species. *Anopheles funestus* had the highest levels of sugar feeding, while *An. coustani* had the lowest level of sugar feeding. The proportion of sugar-fed *An. gambiae* was intermediate. Sugar feeding by males was consistently higher than females within each species. Lower levels of sugar feeding were observed in blood-fed females compared to non-blood females, while levels of sugar feeding among gravid females varied between the different species. Male mosquitoes collected using the aspiration method had higher sugar feeding rates than those from the UV light trap collections. Overall sugar feeding did not vary substantially throughout the night, but the highest intensity of sugar feeding based on the scoring of the cold anthrone test was observed in early evenings and late mornings although it was not possible to determine how long mosquitoes remained positive for sugar in the current experiment.

There have been limited studies of sugar feeding by natural populations of African malaria vectors. In a previous study in two sites in western Kenya, overall sugar feeding rates among *An. gambiae* s.l. and *An. funestus* were 14.4% for host-seeking females and 6.3% for indoor resting females, with no significant differences between the sites or the species^28^. The lack of difference observed between species contrasts with the current study where *An. funestus* was more frequently found sugar-fed compared to *An. gambiae* s.l. Furthermore, the previous study found higher sugar feeding rates among host-seeking females than indoor resting females. The reason for these differences is not clear, although a shift in species composition among *An. gambiae* s.l. from predominantly *An. gambiae* s.s. to predominantly *An. arabiensis* may have contributed to some of the differences observed. In a study in Mali 44.9% of female and 45.1% of male *An. coluzzii* had fed on natural sugar sources based on the cold anthrone test^14^. After attractive sugar bait stations (ASBs) were introduced, sugar feeding on natural sources declined. The proportion that had fed on natural sources was lower than the proportion that had fed on ASBs suggesting that in that setting, natural sugar sources did not interfere with feeding on the artificial bait stations^14^.

There were clear differences in sugar feeding by species in the current study, with the highest overall feeding rates among *An. funestus* and lowest rates among *An. coustani* for both males and females. Compared to other species tested in previous studies, sugar feeding rates among the three *Anopheles* species in the current study were relatively low. Among host-seeking *An. freeborni* in California, 23% were sugar-fed while 36% of non-blood fed, resting females collected in the evening and 55% of non-blood-fed, resting females collected in the morning were sugar-fed. In stark contrast to the current study, 94% of gravid *An. freeborni* were sugar-fed^29^. Feeding rates among *Culex quinquefaciatus* in Texas, as measured by the hot anthrone test, were 60% for males and 72.3% for females^30^, while sugar feeding rates among male and female *Aedes albopictus* in New York were 49.6% and 41.8%, respectively^31^. Variable sugar feeding rates have been observed in *Aedes aegypti,* with high rates observed in Texas^30^ and Mali^32^, while others report little to no sugar feeding by adult female *Ae. aegypti*^33–36^. As noted above, approximately 45% of male and female *An. coluzzii* were sugar fed by the cold anthrone test in Mali^14^.

Based on the low feeding rates observed in some field studies^33,37^ and laboratory studies^34,38,39^, *Ae. aegypti* were thought to derive most of their energetic needs from frequent blood feeding. Similarly, laboratory studies suggested that blood-feeding alone is adequate to meet the nutritional and energetic needs of *An. gambiae* s.s.^40^. The relatively low sugar feeding rates observed in the current study, particularly among blood-fed females, suggest wild *An. gambiae* s.l. and *An. funestus* are also able to meet their energetic needs largely through blood-feeding. These two species, along with *Ae. aegypti*, share similar behavioural traits that may lead to reduced sugar feeding and increased reliance on blood. These species tend to be associated with the domestic and peri-domestic environment and preferentially feed on humans. *Aedes aegypti* has been shown to have increased fitness when fed on human blood alone compared to human blood plus sugar or mouse blood, with or without access to sugar^34^. The difference was attributed to low isoleucine levels in human blood, as supplementing blood meals with this amino acid resulted in lower energetic reserves and lower egg output. It is possible that *An. gambiae* s.s. and *An. funestus,* which feed preferentially on humans, have evolved similar mechanisms to utilise blood meals, although this has not been investigated. However, observations from the current study do not entirely support this hypothesis as the highest prevalence of sugar feeding was in *An. funestus* which has a strong preference for humans, particularly compared to *An. coustani* which feeds on humans at much lower frequencies than *An. funestus* or *An. gambiae* s.l. Furthermore, by molecular identification, *An. gambiae* s.l. were mostly *An. arabiensis* which has emerged as the predominant species in the *An. gambiae* complex in this region^18^. In contrast to *An. funestus* or *An. gambiae* s.s., *An. arabiensis* tends to be much more opportunistic in its feeding behaviours and frequently feeds on cattle, as evidenced by blood meal identification studies^41,42^ and the generally lower sporozoite rates compared to *An. funestus*^19,43,44^.

Sugar feeding is an important aspect of mosquito behaviour that is poorly understood. Differences in rates of sugar feeding by collection method, time of collection and mosquito physiological state may provide insights into this largely unstudied aspect of mosquito biology. Resting male *An. gambiae* s.l., collected by aspiration, were more likely to be sugar-fed than those that were actively flying and captured in UV light traps. Higher rates of sugar feeding in resting males and females have been observed in *Ae. aegypti* and *Cx. quinquefaciatus*^30^, although sample sizes were relatively small and the only statistically significant difference was for *Cx. quinquefaciatus* females. Differences in the prevalence of sugar feeding based on the method of collection have also been observed for *Culex tarsalis*^45^. In *An. freeborni*, male mosquitoes were thought to feed after swarming in the evening and then seek out resting sites while sugars are converted to glycogen^46^. Similar patterns were seen among female *An. freeborni*^29^. The higher levels of sugar feeding among resting *An. gambiae* s.l. and *An. funestus* males suggest similar patterns of sugar feeding followed by resting. However, there was little evidence of temporal variation in sugar feeding among either males or female *An. gambiae* s.l. or *An. funestus*. The lack of variation by time may be partly due to the slow digestion of sugars. However, little is known about rates of digestion of sugar meals in *An. gambiae* or *An. funestus*, particularly among natural populations in western Kenya and interpretation of the proportion sugar fed by time of night should be interpreted with caution. In other species, sugar meals have been observed to be digested within 24 h^31,47^ while at least one study reported detection of sugar in *Ae. aegypti* up to 4 days after ingestion. Slow digestion may have obscured temporal patterns of overall sugar feeding, but the intensity of the cold anthrone results may be indicative of sugar feeding patterns. High intensity results were primarily observed from outdoor collections in the early evening (5–9 pm) for *An. funestus* males with another peak in high intensity anthrone results from collections in the early morning (3–7 am). A similar though less distinct pattern was observed for *An. funestus* females suggesting sugar feeding in this species occurs before commencing nocturnal activities and again before searching out resting sites during the day.

ATSBs have been proposed as a supplementary vector control intervention for malaria control in sub-Saharan Africa based on studies demonstrating their efficacy against *An. gambiae* s.l. mosquitoes in Mali^14,48^. Modelling studies indicate that the minimum ATSB daily excess mortality (measured by daily ATSB feeding rates) in Mali, western Kenya and western Zambia, to achieve a 30%^49^ reduction in malaria incidence compared to areas without ATSBs, are approximately 2.5%, (unpublished data). Although many mosquitoes would likely continue to feed on natural sources, the present study may provide estimates of the maximum potential of ATSBs in western Kenya. Overall feeding rates by *An. funestus* females were 27.7%, suggesting ATSBs could be targeted against this species. Feeding rates were lower among *An. gambiae* s.l. However, *An. gambiae* s.l. are predominantly *An. arabiensis,* which frequently feeds on non-human hosts and is a less efficient vector than *An. funestus* in western Kenya^18^. However, this study does not indicate how wild mosquitoes in western Kenya will respond to the deployment of ATSBs. In Mali, natural sugar feeding declined after the introduction of ASBs suggesting that artificial sugar sources could compete with natural sources. Furthermore, *An. gambiae* and *Ae. aegypti* increase blood feeding when sugar is withheld^50,51^. If the converse is true, ITNs and ATSBs may be complementary vector control tools as ITNs reduce blood feeding by mosquitoes which may in turn increase the frequency of sugar feeding to meet their energetic needs.

There were some limitations to this study. First, it was conducted over 3 months and therefore did not account for seasonal variation in sugar feeding behaviours described in other species^30^. Furthermore, the rates of sugar meal digestion are unknown in *An. gambiae* or *An. funestus*. Different rates of digestion of the sugar meals could affect comparisons in sugar feeding rates among these mosquitoes. Finally, the method used to assess sugar feeding was the cold anthrone test which provides information on the presence or absence of sugar but is not quantitative and relies on subjective assessment of colour intensity for a relative amount of sugar. A more detailed analysis of quantities of sugar and possibly glycogen, which is produced as sugar is digested, would provide more detailed insights into the sugar feeding behaviours of mosquitoes in western Kenya.

## Conclusions

This study indicates that *Anopheles* mosquitoes of both sexes obtain sugar meals from natural sources during all physiological stages related to blood-feeding and egg maturation, whether they rest indoors or outdoors. These findings offer a potential area to exploit for the control of mosquitoes, particularly with the potential advent of ATSBs for the control of malaria in Africa.

## Supplementary Information

Supplementary Information.

## Data Availability

All data generated or analysed during this study are in this published article.

## Abbreviations

LLINs: Long lasting insecticidal nets
IRS: Indoor residual spraying
SSA: Sub Saharan Africa
ATSB: Attractive targeted sugar baits
ASB: Attractive sugar bait
KEMRI: Kenya Medical Research Institute
CDC: Centers for Disease Control and Prevention
HDSS: Health and demographic surveillance system
ITN: Insecticide treated bed net
SERU: Scientific and ethics review unit
UVLT-C: Ultraviolet light trap, close to a house or structure
UVLT-D: Ultraviolet light trap, distant from a house or structure
CDC-LT: CDC light trap
