# Supplementary material for: Natural sugar feeding rates of Anopheles mosquitoes collected by different methods in western Kenya
Source: Sci Rep. 2022 Nov 29;12:20596. doi: 10.1038/s41598-022-25004-9 (PMC9709062; doi:10.1038/s41598-022-25004-9)
Supplement: Supplementary file 1 — Supplementary Information. [file 41598_2022_25004_MOESM1_ESM.docx]

**Supplementary Table 1a.** Full logistic regression model of sugar feeding by species and sex where the outcome is any sugar feeding (anthrone result = 1, 2, or 3) versus no sugar feeding (anthrone result = 0).

| **Parameter** | | **Level** | **Odds Ratio** | **LowerCL** | **UpperCL** | **Z** | **P-Value** |
| --- | --- | --- | --- | --- | --- | --- | --- |
| Intercept |  | | 0.398 | 0.286 | 0.554 | -5.450 | <0.001 |
|  | *An. coustani* | | 0.329 | 0.226 | 0.477 | -5.846 | <0.001 |
| Species | *An. funestus* | | 1.791 | 1.241 | 2.584 | 3.116 | 0.002 |
|  | *An. gambiae* | | Ref. | Ref. | Ref. |  |  |
| Sex | Female | | 0.384 | 0.264 | 0.558 | -5.007 | <0.001 |
|  | Male | | Ref. | Ref. | Ref. |  |  |
| Species * Sex | *An. coustani* * Female | | 2.135 | 1.421 | 3.210 | 3.649 | <0.001 |
|  | *An. coustani* * Male | | Ref. | Ref. | Ref. |  |  |
|  | *An. funestus* * Female | | 1.535 | 1.007 | 2.341 | 1.991 | 0.046 |
|  | *An. funestus* * Male | | Ref. | Ref. | Ref. |  |  |
|  | *An. gambiae* * Female | | Ref. | Ref. | Ref. |  |  |
|  | *An. gambiae* * Male | | Ref. | Ref. | Ref. |  |  |

**Supplementary Table 1b.** Logistic regression pairwise comparisons of sugar feeding by species and sex where the outcome is any sugar feeding (anthrone result = 1, 2, or 3) versus no sugar feeding (anthrone result =0).

| **Species/Sex** | **Method**  **Estimate** | **Reference** | **Reference**  **Estimate** | **Odds Ratio** | **LowerCL** | **UpperCL** | **χ^2^** | **P-Value** |
| --- | --- | --- | --- | --- | --- | --- | --- | --- |
| Female *An. coustani* | 8.3 (7.4-9.2) | Female *An. funestus* | 27.7 (25.0-30.3) | 0.255 | 0.218 | 0.299 | -16.792 | <0.001 |
| Female *An. coustani* | 8.3 (7.4-9.2) | Female *An. gambiae* | 12.8 (10.0-15.7) | 0.702 | 0.593 | 0.831 | -4.115 | <0.001 |
| Female *An. funestus* | 27.7 (25.0-30.3) | Female *An. gambiae* | 12.8 (10.0-15.7) | 2.749 | 2.244 | 3.369 | 9.753 | <0.001 |
| Male *An. coustani* | 9.7 (7.9-11.5) | Male *An. funestus* | 41.3 (34.0-48.6) | 0.184 | 0.137 | 0.247 | -11.219 | <0.001 |
| Male *An. coustani* | 9.7 (7.9-11.5) | Male *An. gambiae* | 27.2 (18.7-35.7) | 0.329 | 0.226 | 0.477 | -5.846 | <0.001 |
| Male *An. funestus* | 41.3 (34.0-48.6) | Male *An. gambiae* | 27.2 (18.7-35.7) | 1.791 | 1.241 | 2.584 | 3.116 | 0.002 |

**Supplementary Table 2a.** Full logistic regression model of sugar feeding by *An. funestus* based on sex/abdominal status where the outcome is any sugar feeding (anthrone result = 1, 2, or 3) versus no sugar feeding (anthrone result = 0).

| **Parameter** | **Odds Ratio** | **LowerCL** | **UpperCL** | **Z** | **P-Value** |
| --- | --- | --- | --- | --- | --- |
| Intercept | 0.61 | 0.54 | 0.7 | -7.327 | <0.001 |
| Blood Unfed | 0.37 | 0.25 | 0.53 | -5.261 | <0.001 |
| Gravid | 0.41 | 0.29 | 0.58 | -5.009 | <0.001 |
| Male | 1.22 | 0.96 | 1.56 | 1.623 | 0.105 |
| Non-Blood Unfed | Ref. | Ref. | Ref. |  |  |

**Supplementary Table 2b.** Logistic regression pairwise comparisons of sugar feeding by *An. funestus* based on sex/abdominal status where the outcome is any sugar feeding (anthrone result = 1, 2, or 3) versus no sugar feeding (anthrone result =0).

| **Status** | **Status**  **Estimate** | **Reference** | **Reference**  **Estimate** | **Odds Ratio** | **LowerCL** | **UpperCL** | **χ^2^** | **P-Value** |
| --- | --- | --- | --- | --- | --- | --- | --- | --- |
| Blood Fed | 21.9 (17.9-26) | Gravid | 22.3 (17.6-27) | 0.903 | 0.618 | 1.318 | 0.281 | 0.596 |
| Blood Fed | 21.9 (17.9-26) | Male | 41.3 (34-48.6) | 0.301 | 0.199 | 0.454 | 32.534 | <0.001 |
| Gravid | 22.3 (17.6-27) | Male | 41.3 (34-48.6) | 0.333 | 0.228 | 0.488 | 31.910 | <0.001 |
| Blood Fed | 21.9 (17.9-26) | Non-Blood Fed | 30.4 (27.2-33.5) | 0.368 | 0.253 | 0.534 | 27.679 | <0.001 |
| Gravid | 22.3 (17.6-27) | Non-Blood Fed | 30.4 (27.2-33.5) | 0.407 | 0.287 | 0.579 | 25.093 | <0.001 |
| Male | 41.3 (34-48.6) | Non-Blood Fed | 30.4 (27.2-33.5) | 1.222 | 0.959 | 1.558 | 2.633 | 0.105 |

**Supplementary Table 3a.** Full logistic regression model of sugar feeding by *An. gambiae* based on sex/abdominal status where the outcome is any sugar feeding (anthrone result = 1, 2, or 3) versus no sugar feeding (anthrone result = 0).

| **Parameter** | **Odds Ratio** | **LowerCL** | **UpperCL** | **Z** | **P-Value** |
| --- | --- | --- | --- | --- | --- |
| Intercept | 0.15 | 0.13 | 0.18 | -19.148 | <0.001 |
| Blood Fed | 0.34 | 0.12 | 0.94 | -2.07 | 0.038 |
| Gravid | 0.78 | 0.36 | 1.68 | -0.638 | 0.523 |
| Male | 2.56 | 1.75 | 3.74 | 4.862 | <0.001 |
| Non-Blood Unfed | Ref. | Ref. | Ref. |  |  |

**Supplementary Table 3b.** Logistic regression pairwise comparisons of sugar feeding by *An. gambiae* based on sex/abdominal status where the outcome is any sugar feeding (anthrone result = 1, 2, or 3) versus no sugar feeding (anthrone result =0).

| **Status** | **Status**  **Estimate** | **Reference** | **Reference**  **Estimate** | **Odds Ratio** | **LowerCL** | **UpperCL** | **χ^2^** | **P-Value** |
| --- | --- | --- | --- | --- | --- | --- | --- | --- |
| Blood Fed | 5.3 (0.6-10.1) | Gravid | 10.0 (2.8-17.2) | 0.432 | 0.125 | 1.492 | 1.762 | 0.184 |
| Blood Fed | 5.3 (0.6-10.1) | Male | 27.2 (18.7-35.7) | 0.131 | 0.044 | 0.390 | 13.346 | <0.001 |
| Gravid | 10.0 (2.8-17.2) | Male | 27.2 (18.7-35.7) | 0.304 | 0.127 | 0.732 | 7.066 | 0.008 |
| Blood Fed | 5.3 (0.6-10.1) | Non-Blood Fed | 13.4 (10.4-16.5) | 0.336 | 0.120 | 0.944 | 4.283 | 0.038 |
| Gravid | 10.0 (2.8-17.2) | Non-Blood Fed | 13.4 (10.4-16.5) | 0.779 | 0.362 | 1.677 | 0.407 | 0.523 |
| Male | 27.2 (18.7-35.7) | Non-Blood Fed | 13.4 (10.4-16.5) | 2.561 | 1.753 | 3.741 | 23.642 | <0.001 |

**Supplementary Table 4a.** Full logistic regression model of sugar feeding by *An. coustani* based on sex/abdominal status where the outcome is any sugar feeding (anthrone result = 1, 2, or 3) versus no sugar feeding (anthrone result = 0).

| **Parameter** | **Odds Ratio** | **LowerCL** | **UpperCL** | **Z** | **P-Value** |
| --- | --- | --- | --- | --- | --- |
| Intercept | 0.10 | 0.09 | 0.12 | -41.037 | <0.001 |
| Blood Unfed | 0.56 | 0.41 | 0.77 | -3.543 | <0.001 |
| Gravid | 0.92 | 0.36 | 2.33 | -0.183 | 0.855 |
| Male | 1.19 | 0.99 | 1.43 | 1.892 | 0.058 |
| Non-Blood Unfed | Ref. | Ref. | Ref. |  |  |

**Supplementary Table 4b.** Logistic regression pairwise comparisons of sugar feeding by *An. coustani* based on sex/abdominal status where the outcome is any sugar feeding (anthrone result = 1, 2, or 3) versus no sugar feeding (anthrone result =0).

| **Status** | **Status**  **Estimate** | **Reference** | **Reference**  **Estimate** | **Odds Ratio** | **LowerCL** | **UpperCL** | **χ^2^** | **P-Value** |
| --- | --- | --- | --- | --- | --- | --- | --- | --- |
| Blood Fed | 4.9 (3.3-6.5) | Gravid | 8.5 (0.9-16.1) | 0.615 | 0.233 | 1.625 | 0.962 | 0.327 |
| Blood Fed | 4.9 (3.3-6.5) | Male | 9.7 (7.9-11.5) | 0.473 | 0.331 | 0.676 | 16.869 | <0.001 |
| Gravid | 8.5 (0.9-16.1) | Male | 9.7 (7.9-11.5) | 0.770 | 0.299 | 1.980 | 0.295 | 0.587 |
| Blood Fed | 4.9 (3.3-6.5) | Non-Blood Fed | 8.6 (7.6-9.6) | 0.564 | 0.410 | 0.774 | 12.551 | <0.001 |
| Gravid | 8.5 (0.9-16.1) | Non-Blood Fed | 8.6 (7.6-9.6) | 0.917 | 0.361 | 2.328 | 0.034 | 0.855 |
| Male | 9.7 (7.9-11.5) | Non-Blood Fed | 8.6 (7.6-9.6) | 1.191 | 0.994 | 1.428 | 3.581 | 0.058 |

**Supplementary Table 5a.** Full logistic regression model of sugar feeding by *An. funestus* females based on collection method where the outcome is any sugar feeding (anthrone result = 1, 2, or 3) versus no sugar feeding (anthrone result = 0).

| **Parameter** | **Odds Ratio** | **LowerCL** | **UpperCL** | **Z** | **P-Value** |
| --- | --- | --- | --- | --- | --- |
| Intercept | 0.44 | 0.32 | 0.61 | -4.997 | <0.001 |
| Aspiration Indoor | 1.04 | 0.72 | 1.52 | 0.215 | 0.829 |
| Aspiration Outdoor | 0.80 | 0.39 | 1.61 | -0.629 | 0.529 |
| Malaise | 0.75 | 0.22 | 2.55 | -0.456 | 0.648 |
| UVLT Indoor | 0.81 | 0.56 | 1.18 | -1.11 | 0.267 |
| UVLT Outdoor-C | 1.00 | 0.67 | 1.49 | -0.012 | 0.991 |
| UVLT Outdoor-D | Ref. | Ref. | Ref. |  |  |

**Supplementary Table 5b.** Logistic regression pairwise comparisons of sugar feeding by *An. funestus* females based on collection method where the outcome is any sugar feeding (anthrone result = 1, 2, or 3) versus no sugar feeding (anthrone result =0).

| **Method** | **Method**  **Estimate** | **Reference** | **Reference**  **Estimate** | **Odds Ratio** | **LowerCL** | **UpperCL** | **χ^2^** | **P-Value** |
| --- | --- | --- | --- | --- | --- | --- | --- | --- |
| Aspiration Indoor | 28.1 (23.4-32.9) | Aspiration Outdoor | 26.7 (15.4-38) | 1.306 | 0.674 | 2.531 | 0.626 | 0.429 |
| Aspiration Indoor | 28.1 (23.4-32.9) | Malaise | 25.0 (3.4-46.6) | 1.384 | 0.420 | 4.563 | 0.285 | 0.593 |
| Aspiration Outdoor | 26.7 (15.4-38) | Malaise | 25.0 (3.4-46.6) | 1.06 | 0.279 | 4.020 | 0.007 | 0.932 |
| Aspiration Indoor | 28.1 (23.4-32.9) | UVLT Indoor | 26.4 (22-30.8) | 1.287 | 0.990 | 1.673 | 3.564 | 0.059 |
| Aspiration Outdoor | 26.7 (15.4-38) | UVLT Indoor | 26.4 (22-30.8) | 0.986 | 0.506 | 1.920 | 0.002 | 0.966 |
| Malaise | 25.o (3.4-46.6) | UVLT Indoor | 26.4 (22-30.8) | 0.930 | 0.282 | 3.065 | 0.014 | 0.905 |
| Aspiration Indoor | 28.1 (23.4-32.9) | UVLT Outdoor-C | 30.4 (25.3-35.6) | 1.045 | 0.763 | 1.430 | 0.074 | 0.786 |
| Aspiration Outdoor | 26.7 (15.4-38) | UVLT Outdoor-C | 30.4 (25.3-35.6) | 0.800 | 0.408 | 1.568 | 0.423 | 0.516 |
| Malaise | 25.0 (3.4-46.6) | UVLT Outdoor-C | 30.4 (25.3-35.6) | 0.755 | 0.227 | 2.512 | 0.21 | 0.646 |
| UVLT Indoor | 26.4 (22-30.8) | UVLT Outdoor-C | 30.4 (25.3-35.6) | 0.811 | 0.594 | 1.109 | 1.717 | 0.19 |
| Aspiration Indoor | 28.1 (23.4-32.9) | UVLT Outdoor-D | 29.7 (22.7-36.7) | 1.042 | 0.716 | 1.516 | 0.046 | 0.829 |
| Aspiration Outdoor | 26.7 (15.4-38) | UVLT Outdoor-D | 29.7 (22.7-36.7) | 0.798 | 0.395 | 1.613 | 0.396 | 0.529 |
| Malaise | 25.0 (3.4-46.6) | UVLT Outdoor-D | 29.7 (22.7-36.7) | 0.753 | 0.222 | 2.549 | 0.208 | 0.648 |
| UVLT Indoor | 26.4 (22-30.8) | UVLT Outdoor-D | 29.7 (22.7-36.7) | 0.810 | 0.557 | 1.176 | 1.232 | 0.267 |
| UVLT Outdoor-C | 30.4 (25.3-35.6) | UVLT Outdoor-D | 29.7 (22.7-36.7) | 0.998 | 0.669 | 1.487 | <0.001 | 0.991 |

**Supplementary Table 6a.** Full logistic regression model of sugar feeding by *An. funestus* males based on collection method where the outcome is any sugar feeding (anthrone result = 1, 2, or 3) versus no sugar feeding (anthrone result = 0).

| **Parameter** | **Odds Ratio** | **LowerCL** | **UpperCL** | **Z** | **P-Value** |
| --- | --- | --- | --- | --- | --- |
| Intercept | 1.17 | 0.59 | 2.32 | 0.455 | 0.649 |
| Aspiration Indoor | 1.10 | 0.54 | 2.25 | 0.269 | 0.788 |
| Aspiration Outdoor | 1.10 | 0.5 | 2.42 | 0.225 | 0.822 |
| Malaise | 0.37 | 0.06 | 2.29 | -1.075 | 0.282 |
| UVLT Indoor | 0.12 | 0.06 | 0.25 | -5.710 | <0.001 |
| UVLT Outdoor-C | 0.73 | 0.26 | 2.06 | -0.596 | 0.551 |
| UVLT Outdoor-D | Ref. | Ref. | Ref. |  |  |

**Supplementary Table 6b.** Logistic regression pairwise comparisons of sugar feeding by *An. funestus* males based on collection method where the outcome is any sugar feeding (anthrone result = 1, 2, or 3) versus no sugar feeding (anthrone result =0).

| **Method** | **Method**  **Estimate** | **Reference** | **Reference**  **Estimate** | **Odds Ratio** | **LowerCL** | **UpperCL** | **χ^2^** | **P-Value** |
| --- | --- | --- | --- | --- | --- | --- | --- | --- |
| Aspiration Indoor | 56.2 (50.9-61.5) | Aspiration Outdoor | 57.4 (47.6-67.1) | 1.007 | 0.653 | 1.552 | 0.001 | 0.975 |
| Aspiration Indoor | 56.2 (50.9-61.5) | Malaise | 27.3 (0-59) | 3.013 | 0.543 | 16.712 | 1.592 | 0.207 |
| Aspiration Outdoor | 57.4 (47.6-67.1) | Malaise | 27.3 (0-59) | 2.992 | 0.521 | 17.176 | 1.512 | 0.219 |
| Aspiration Indoor | 56.2 (50.9-61.5) | UVLT Indoor | 12.0 (9.5-14.5) | 9.317 | 6.643 | 13.067 | 167.257 | <0.001 |
| Aspiration Outdoor | 57.4 (47.6-67.1) | UVLT Indoor | 12.0 (9.5-14.5) | 9.254 | 5.708 | 15.001 | 81.49 | <0.001 |
| Malaise | 27.3 (0-59) | UVLT Indoor | 12.0 (9.5-14.5) | 3.092 | 0.553 | 17.298 | 1.652 | 0.199 |
| Aspiration Indoor | 56.2 (50.9-61.5) | UVLT Outdoor-C | 46.7 (25.2-68.2) | 1.511 | 0.634 | 3.602 | 0.869 | 0.351 |
| Aspiration Outdoor | 57.4 (47.6-67.1) | UVLT Outdoor-C | 46.7 (25.2-68.2) | 1.501 | 0.590 | 3.82 | 0.726 | 0.394 |
| Malaise | 27.3 (0-59) | UVLT Outdoor-C | 46.7 (25.2-68.2) | 0.502 | 0.075 | 3.348 | 0.508 | 0.476 |
| UVLT Indoor | 12.0 (9.5-14.5) | UVLT Outdoor-C | 46.7 (25.2-68.2) | 0.162 | 0.067 | 0.393 | 16.224 | <0.001 |
| Aspiration Indoor | 56.2 (50.9-61.5) | UVLT Outdoor-D | 53.8 (36.8-70.9) | 1.103 | 0.541 | 2.248 | 0.072 | 0.788 |
| Aspiration Outdoor | 57.4 (47.6-67.1) | UVLT Outdoor-D | 53.8 (36.8-70.9) | 1.095 | 0.496 | 2.416 | 0.051 | 0.822 |
| Malaise | 27.3 (0-59) | UVLT Outdoor-D | 53.8 (36.8-70.9) | 0.366 | 0.059 | 2.286 | 1.157 | 0.282 |
| UVLT Indoor | 12.0 (9.5-14.5) | UVLT Outdoor-D | 53.8 (36.8-70.9) | 0.118 | 0.057 | 0.246 | 32.607 | <0.001 |
| UVLT Outdoor-C | 46.7 (25.2-68.2) | UVLT Outdoor-D | 53.8 (36.8-70.9) | 0.730 | 0.259 | 2.058 | 0.355 | 0.551 |

**Supplementary Table 7a.** Full logistic regression model of sugar feeding by *An. gambiae* females based on collection method where the outcome is any sugar feeding (anthrone result = 1, 2, or 3) versus no sugar feeding (anthrone result = 0).

| **Parameter** | **Odds Ratio** | **LowerCL** | **UpperCL** | **Z** | **P-Value** |
| --- | --- | --- | --- | --- | --- |
| Intercept | 0.14 | 0.10 | 0.19 | -12.125 | <0.001 |
| Aspiration Indoor | 0.77 | 0.36 | 1.64 | -0.672 | 0.501 |
| Aspiration Outdoor | 1.79 | 0.78 | 4.08 | 1.376 | 0.169 |
| Malaise | 1.63 | 0.57 | 4.67 | 0.907 | 0.364 |
| UVLT Indoor | 1.06 | 0.64 | 1.77 | 0.241 | 0.81 |
| UVLT Outdoor-C | 0.92 | 0.63 | 1.37 | -0.392 | 0.695 |
| UVLT Outdoor-D | Ref. | Ref. | Ref. |  |  |

**Supplementary Table 7b.** Logistic regression pairwise comparisons of sugar feeding by *An. gambiae* females based on collection method where the outcome is any sugar feeding (anthrone result = 1, 2, or 3) versus no sugar feeding (anthrone result =0).

| **Method** | **Method**  **Estimate** | **Reference** | **Reference**  **Estimate** | **Odds Ratio** | **LowerCL** | **UpperCL** | **χ^2^** | **P-Value** |
| --- | --- | --- | --- | --- | --- | --- | --- | --- |
| Aspiration Indoor | 10.0 (3.7-16.3) | Aspiration Outdoor | 20.0 (9.2-30.8) | 0.433 | 0.156 | 1.199 | 2.595 | 0.107 |
| Aspiration Indoor | 10.0 (3.7-16.3) | Malaise | 18.2 (3.8-32.6) | 0.474 | 0.141 | 1.595 | 1.452 | 0.228 |
| Aspiration Outdoor | 20.0 (9.2-30.8) | Malaise | 18.2 (3.8-32.6) | 1.097 | 0.311 | 3.868 | 0.021 | 0.886 |
| Aspiration Indoor | 10.0 (3.7-16.3) | UVLT Indoor | 11.6 (7-16.3) | 0.726 | 0.335 | 1.571 | 0.661 | 0.416 |
| Aspiration Outdoor | 20.0 (9.2-30.8) | UVLT Indoor | 11.6 (7-16.3) | 1.678 | 0.713 | 3.948 | 1.405 | 0.236 |
| Malaise | 18.2 (3.8-32.6) | UVLT Indoor | 11.6 (7-16.3) | 1.530 | 0.521 | 4.493 | 0.599 | 0.439 |
| Aspiration Indoor | 10.0 (3.7-16.3) | UVLT Outdoor-C | 13.6 (9.1-18.2) | 0.836 | 0.398 | 1.754 | 0.225 | 0.635 |
| Aspiration Outdoor | 20.0 (9.2-30.8) | UVLT Outdoor-C | 13.6 (9.1-18.2) | 1.932 | 0.853 | 4.375 | 2.491 | 0.115 |
| Malaise | 18.2 (3.8-32.6) | UVLT Outdoor-C | 13.6 (9.1-18.2) | 1.761 | 0.618 | 5.019 | 1.123 | 0.289 |
| UVLT Indoor | 11.6 (7-16.3) | UVLT Outdoor-C | 13.6 (9.1-18.2) | 1.151 | 0.705 | 1.880 | 0.317 | 0.574 |
| Aspiration Indoor | 10.0 (3.7-16.3) | UVLT Outdoor-D | 11.8 (8-15.7) | 0.773 | 0.365 | 1.638 | 0.452 | 0.501 |
| Aspiration Outdoor | 20.0 (9.2-30.8) | UVLT Outdoor-D | 11.8 (8-15.7) | 1.786 | 0.782 | 4.081 | 1.893 | 0.169 |
| Malaise | 18.2 (3.8-32.6) | UVLT Outdoor-D | 11.8 (8-15.7) | 1.629 | 0.568 | 4.673 | 0.823 | 0.364 |
| UVLT Indoor | 11.6 (7-16.3) | UVLT Outdoor-D | 11.8 (8-15.7) | 1.064 | 0.640 | 1.770 | 0.058 | 0.810 |
| UVLT Outdoor-C | 13.6 (9.1-18.2) | UVLT Outdoor-D | 11.8 (8-15.7) | 0.925 | 0.625 | 1.367 | 0.154 | 0.695 |

**Supplementary Table 8a.** Full logistic regression model of sugar feeding by *An. gambiae* males based on collection method where the outcome is any sugar feeding (anthrone result = 1, 2, or 3) versus no sugar feeding (anthrone result = 0).

| **Parameter** | **Odds Ratio** | **LowerCL** | **UpperCL** | **Z** | **P-Value** |
| --- | --- | --- | --- | --- | --- |
| Intercept | 0.26 | 0.14 | 0.49 | -4.213 | <0.001 |
| Aspiration Indoor | 4.72 | 1.82 | 12.27 | 3.187 | 0.001 |
| Aspiration Outdoor | 5.72 | 1.91 | 17.16 | 3.11 | 0.002 |
| Malaise | 2.29 | 0.48 | 10.88 | 1.038 | 0.299 |
| UVLT Indoor | 1.55 | 0.52 | 4.65 | 0.784 | 0.433 |
| UVLT Outdoor-C | 0.55 | 0.2 | 1.55 | -1.134 | 0.257 |
| UVLT Outdoor-D | Ref. | Ref. | Ref. |  |  |

**Supplementary Table 8b.** Logistic regression pairwise comparisons of sugar feeding by *An. gambiae* males based on collection method where the outcome is any sugar feeding (anthrone result = 1, 2, or 3) versus no sugar feeding (anthrone result =0).

| **Method** | **Method**  **Estimate** | **Reference** | **Reference**  **Estimate** | **Odds Ratio** | **LowerCL** | **UpperCL** | **χ^2^** | **P-Value** |
| --- | --- | --- | --- | --- | --- | --- | --- | --- |
| Aspiration Indoor | 55.9 (38.3-73.4) | Aspiration Outdoor | 60.7 (41.2-80.2) | 0.826 | 0.243 | 2.807 | 0.094 | 0.759 |
| Aspiration Indoor | 55.9 (38.3-73.4) | Malaise | 37.5 (3.9-71.1) | 2.067 | 0.416 | 10.279 | 0.787 | 0.375 |
| Aspiration Outdoor | 60.7 (41.2-80.2) | Malaise | 37.5 (3.9-71.1) | 2.502 | 0.460 | 13.615 | 1.126 | 0.289 |
| Aspiration Indoor | 55.9 (38.3-73.4) | UVLT Indoor | 16.4 (0.9-31.8) | 3.044 | 1.028 | 9.014 | 4.038 | 0.044 |
| Aspiration Outdoor | 60.7 (41.2-80.2) | UVLT Indoor | 16.4 (0.9-31.8) | 3.685 | 1.022 | 13.287 | 3.974 | 0.046 |
| Malaise | 37.5 (3.9-71.1) | UVLT Indoor | 16.4 (0.9-31.8) | 1.473 | 0.271 | 8.008 | 0.201 | 0.654 |
| Aspiration Indoor | 55.9 (38.3-73.4) | UVLT Outdoor-C | 12.5 (4.7-20.3) | 8.587 | 2.933 | 25.141 | 15.391 | <0.001 |
| Aspiration Outdoor | 60.7 (41.2-80.2) | UVLT Outdoor-C | 12.5 (4.7-20.3) | 10.397 | 3.119 | 34.665 | 14.525 | <0.001 |
| Malaise | 37.5 (3.9-71.1) | UVLT Outdoor-C | 12.5 (4.7-20.3) | 4.155 | 0.809 | 21.346 | 2.910 | 0.088 |
| UVLT Indoor | 16.4 (0.9-31.8) | UVLT Outdoor-C | 12.5 (4.7-20.3) | 2.821 | 0.847 | 9.398 | 2.854 | 0.091 |
| Aspiration Indoor | 55.9 (38.3-73.4) | UVLT Outdoor-D | 20.3 (10.4-30.2) | 4.723 | 1.818 | 12.270 | 10.158 | 0.001 |
| Aspiration Outdoor | 60.7 (41.2-80.2) | UVLT Outdoor-D | 20.3 (10.4-30.2) | 5.719 | 1.906 | 17.160 | 9.673 | 0.002 |
| Malaise | 37.5 (3.9-71.1) | UVLT Outdoor-D | 20.3 (10.4-30.2) | 2.285 | 0.480 | 10.883 | 1.077 | 0.299 |
| UVLT Indoor | 16.4 (0.9-31.8) | UVLT Outdoor-D | 20.3 (10.4-30.2) | 1.552 | 0.518 | 4.652 | 0.615 | 0.433 |
| UVLT Outdoor-C | 12.5 (4.7-20.3) | UVLT Outdoor-D | 20.3 (10.4-30.2) | 0.550 | 0.196 | 1.546 | 1.285 | 0.257 |

**Supplementary Table 9a.** Full logistic regression model of sugar feeding by *An. coustani* females based on collection method where the outcome is any sugar feeding (anthrone result = 1, 2, or 3) versus no sugar feeding (anthrone result = 0).

| **Parameter** | **Odds Ratio** | **LowerCL** | **UpperCL** | **Z** | **P-Value** |
| --- | --- | --- | --- | --- | --- |
| Intercept | 0.10 | 0.09 | 0.12 | -29.739 | <0.001 |
| Aspiration Indoor | 7.76 | 2.01 | 29.91 | 2.976 | 0.003 |
| Aspiration Outdoor | 1.33 | 0.29 | 6.20 | 0.363 | 0.716 |
| Malaise | 1.22 | 0.61 | 2.45 | 0.568 | 0.570 |
| UVLT Indoor | 0.54 | 0.27 | 1.07 | -1.767 | 0.077 |
| UVLT Outdoor-C | 0.97 | 0.77 | 1.23 | -0.237 | 0.813 |
| UVLT Outdoor-D | Ref. | Ref. | Ref. |  |  |

**Supplementary Table 9b.** Full logistic regression model of sugar feeding by *An. coustani* females based on collection method where the outcome is any sugar feeding (anthrone result = 1, 2, or 3) versus no sugar feeding (anthrone result = 0).

| **Method** | **Method**  **Estimate** | **Reference** | **Reference**  **Estimate** | **Odds Ratio** | **LowerCL** | **UpperCL** | **χ^2^** | **P-Value** |
| --- | --- | --- | --- | --- | --- | --- | --- | --- |
| Aspiration Indoor | 40.0 (6.7-73.3) | Aspiration Outdoor | 11.1 (0-26.4) | 5.834 | 0.762 | 44.676 | 2.883 | 0.09 |
| Aspiration Indoor | 40.0 (6.7-73.3) | Malaise | 10.9 (4.3-17.5) | 6.344 | 1.412 | 28.509 | 5.807 | 0.016 |
| Aspiration Outdoor | 11.1 (0-26.4) | Malaise | 10.9 (4.3-17.5) | 1.087 | 0.204 | 5.809 | 0.010 | 0.922 |
| Aspiration Indoor | 40.0 (6.7-73.3) | UVLT Indoor | 5.0 (2.7-7.3) | 14.353 | 3.800 | 54.216 | 15.434 | <0.001 |
| Aspiration Outdoor | 11.1 (0-26.4) | UVLT Indoor | 5.0 (2.7-7.3) | 2.460 | 0.463 | 13.072 | 1.116 | 0.291 |
| Malaise | 10.9 (4.3-17.5) | UVLT Indoor | 5.0 (2.7-7.3) | 2.262 | 0.875 | 5.851 | 2.835 | 0.092 |
| Aspiration Indoor | 40.0 (6.7-73.3) | UVLT Outdoor-C | 8.6 (7.3-10) | 7.984 | 2.066 | 30.849 | 9.073 | 0.003 |
| Aspiration Outdoor | 11.1 (0-26.4) | UVLT Outdoor-C | 8.6 (7.3-10) | 1.368 | 0.293 | 6.394 | 0.159 | 0.690 |
| Malaise | 10.9 (4.3-17.5) | UVLT Outdoor-C | 8.6 (7.3-10) | 1.258 | 0.625 | 2.535 | 0.414 | 0.520 |
| UVLT Indoor | 5.0 (2.7-7.3) | UVLT Outdoor-C | 8.6 (7.3-10) | 0.556 | 0.280 | 1.106 | 2.800 | 0.094 |
| Aspiration Indoor | 40.0 (6.7-73.3) | UVLT Outdoor-D | 8.3 (7-9.5) | 7.760 | 2.013 | 29.911 | 8.858 | 0.003 |
| Aspiration Outdoor | 11.1 (0-26.4) | UVLT Outdoor-D | 8.3 (7-9.5) | 1.330 | 0.285 | 6.202 | 0.132 | 0.716 |
| Malaise | 10.9 (4.3-17.5) | UVLT Outdoor-D | 8.3 (7-9.5) | 1.223 | 0.610 | 2.452 | 0.322 | 0.570 |
| UVLT Indoor | 5.0 (2.7-7.3) | UVLT Outdoor-D | 8.3 (7-9.5) | 0.541 | 0.273 | 1.070 | 3.121 | 0.077 |
| UVLT Outdoor-C | 8.6 (7.3-10) | UVLT Outdoor-D | 8.3 (7-9.5) | 0.972 | 0.768 | 1.230 | 0.056 | 0.813 |

**Supplementary Table 10a.** Full logistic regression model of sugar feeding by *An. coustani* males based on collection method where the outcome is any sugar feeding (anthrone result = 1, 2, or 3) versus no sugar feeding (anthrone result = 0).

| **Parameter** | **Odds Ratio** | **LowerCL** | **UpperCL** | **Z** | **P-Value** |
| --- | --- | --- | --- | --- | --- |
| Intercept | 0.10 | 0.08 | 0.13 | -17.054 | <0.001 |
| Malaise | 1.10 | 0.13 | 9.17 | 0.084 | 0.933 |
| UVLT Indoor | 0.53 | 0.07 | 4.00 | -0.616 | 0.538 |
| UVLT Outdoor-C | 1.39 | 0.95 | 2.01 | 1.713 | 0.087 |
| UVLT Outdoor-D | Ref. | Ref. | Ref. |  |  |

**Supplementary Table 10b.** Full logistic regression model of sugar feeding by *An. coustani* males based on collection method where the outcome is any sugar feeding (anthrone result = 1, 2, or 3) versus no sugar feeding (anthrone result = 0).

| **Method** | **Method**  **Estimate** | **Reference** | **Reference**  **Estimate** | **Odds Ratio** | **LowerCL** | **UpperCL** | **χ^2^** | **P-Value** |
| --- | --- | --- | --- | --- | --- | --- | --- | --- |
| Malaise | 10.0 (0-29) | UVLT Indoor | 3.6 (0-11.4) | 2.069 | 0.113 | 38.008 | 0.240 | 0.624 |
| Malaise | 10.0 (0-29) | UVLT Outdoor-C | 12.1 (9.1-15) | 0.790 | 0.094 | 6.624 | 0.047 | 0.828 |
| UVLT Indoor | 3.6 (0-11.4) | UVLT Outdoor-C | 12.1 (9.1-15) | 0.382 | 0.050 | 2.893 | 0.869 | 0.351 |
| Malaise | 10.0 (0-29) | UVLT Outdoor-D | 8.7 (6.5-10.8) | 1.095 | 0.131 | 9.166 | 0.007 | 0.933 |
| UVLT Indoor | 3.6 (0-11.4) | UVLT Outdoor-D | 8.7 (6.5-10.8) | 0.529 | 0.070 | 4.002 | 0.380 | 0.538 |
| UVLT Outdoor-C | 12.1 (9.1-15) | UVLT Outdoor-D | 8.7 (6.5-10.8) | 1.386 | 0.954 | 2.015 | 2.935 | 0.087 |

**Supplementary Table 11a.** Full logistic regression model of sugar feeding by *An. funestus* females based on time of collection where the outcome is any sugar feeding (anthrone result = 1, 2, or 3) versus no sugar feeding (anthrone result = 0).

| **Parameter** | | **Odds Ratio** | **LowerCL** | | **UpperCL** | | **Z** | | **P-Value** | |  |
| --- | --- | --- | --- | --- | --- | --- | --- | --- | --- | --- | --- |
| Interceptor | 0.46 | | | 0.29 | | 0.73 | | -3.306 | | 0.001 | |
| Early Evening (5pm to 9pm) | 0.90 | | | 0.50 | | 1.60 | | -0.372 | | 0.710 | |
| Late Evening (9pm to 12am) | 0.70 | | | 0.38 | | 1.29 | | -1.156 | | 0.248 | |
| Middle of Night (12am to 3am) | 1.32 | | | 0.73 | | 2.41 | | 0.917 | | 0.359 | |
| Early Morning (3am to 7am) | Ref. | | | Ref. | | Ref. | |  | |  | |

**Supplementary Table 11b.** Full logistic regression model of sugar feeding by *An. funestus* females based on time of collection where the outcome is any sugar feeding (anthrone result = 1, 2, or 3) versus no sugar feeding (anthrone result = 0).

| **Collection Time** | **Collection Time**  **Estimate** | **Reference** | **Reference**  **Estimate** | **Odds Ratio** | **LowerCL** | **UpperCL** | **χ^2^** | **P-Value** |
| --- | --- | --- | --- | --- | --- | --- | --- | --- |
| Early Evening  (5pm to 9pm) | 28.9 (21.7-36.2) | Early Morning  (3am to 7am) | 31.1 (20.9-41.2) | 0.896 | 0.501 | 1.601 | 0.139 | 0.710 |
| Early Evening  (5pm to 9pm) | 28.9 (21.7-36.2) | Late Evening  (9pm to 12am) | 23.6 (16.2-31) | 1.285 | 0.757 | 2.18 | 0.862 | 0.353 |
| Early Evening  (5pm to 9pm) | 28.9 (21.7-36.2) | Middle of Night  (12am to 3am) | 37.5 (28.7-46.3) | 0.677 | 0.405 | 1.131 | 2.221 | 0.136 |
| Late Evening  (9pm to 12am) | 23.6 (16.2-31) | Early Morning  (3am to 7am) | 31.1 (20.9-41.2) | 0.697 | 0.378 | 1.285 | 1.336 | 0.248 |
| Late Evening  (9pm to 12am) | 23.6 (16.2-31) | Middle of Night  (12am to 3am) | 37.5 (28.7-46.3) | 0.527 | 0.304 | 0.912 | 5.247 | **0.022** |
| Middle of Night  (12am to 3am) | 37.5 (28.7-46.3) | Early Morning  (3am to 7am) | 31.1 (20.9-41.2) | 1.323 | 0.727 | 2.408 | 0.840 | 0.359 |

**Supplementary Table 12a.** Full logistic regression model of sugar feeding by *An. funestus* males based on time of collection where the outcome is any sugar feeding (anthrone result = 1, 2, or 3) versus no sugar feeding (anthrone result = 0).

| **Parameter** | | **Odds Ratio** | **LowerCL** | | **UpperCL** | | **Z** | | **P-Value** | |  |
| --- | --- | --- | --- | --- | --- | --- | --- | --- | --- | --- | --- |
| Interceptor | 0.46 | | | 0.12 | | 1.77 | | -1.132 | | 0.258 | |
| Early Evening (5pm to 9pm) | 2.43 | | | 0.54 | | 11.07 | | 1.151 | | 0.250 | |
| Late Evening (9pm to 12am) | 2.18 | | | 0.35 | | 13.69 | | 0.834 | | 0.404 | |
| Middle of Night (12am to 3am) | 4.37 | | | 0.28 | | 68.73 | | 1.049 | | 0.294 | |
| Early Morning (3am to 7am) | Ref. | | | Ref. | | Ref. | |  | |  | |

**Supplementary Table 12b.** Full logistic regression model of sugar feeding by *An. funestus* males based on time of collection where the outcome is any sugar feeding (anthrone result = 1, 2, or 3) versus no sugar feeding (anthrone result = 0).

| **Collection Time** | **Collection Time**  **Estimate** | **Reference** | **Reference**  **Estimate** | **Odds Ratio** | **LowerCL** | **UpperCL** | **χ^2^** | **P-Value** |
| --- | --- | --- | --- | --- | --- | --- | --- | --- |
| Early Evening  (5pm to 9pm) | 55.8*(37.2-74.4) | Early Morning (3am to 7am) | 30*(6.2-53.8) | 2.434 | 0.535 | 11.065 | 1.325 | 0.250 |
| Early Evening  (5pm to 9pm) | 55.8*(37.2-74.4) | Late Evening (9pm to 12am) | 50*(17.9-82.1) | 1.114 | 0.271 | 4.58 | 0.022 | 0.881 |
| Early Evening  (5pm to 9pm) | 55.8*(37.2-74.4) | Middle of Night (12am to 3am) | 66.7*(11.5-100) | 0.557 | 0.046 | 6.751 | 0.211 | 0.646 |
| Late Evening  (9pm to 12am) | 50*(17.9-82.1) | Early Morning (3am to 7am) | 30*(6.2-53.8) | 2.184 | 0.349 | 13.689 | 0.696 | 0.404 |
| Late Evening  (9pm to 12am) | 50*(17.9-82.1) | Middle of Night (12am to 3am) | 66.7*(11.5-100) | 0.5 | 0.034 | 7.452 | 0.253 | 0.615 |
| Middle of Night  (12am to 3am) | 66.7*(11.5-100) | Early Morning (3am to 7am) | 30*(6.2-53.8) | 4.369 | 0.278 | 68.727 | 1.100 | 0.294 |

**Supplementary Table 13a.** Full logistic regression model of sugar feeding by *An. gambiae* females based on time of collection where the outcome is any sugar feeding (anthrone result = 1, 2, or 3) versus no sugar feeding (anthrone result = 0).

| **Parameter** | | **Odds Ratio** | **LowerCL** | | **UpperCL** | | **Z** | | **P-Value** | |  |
| --- | --- | --- | --- | --- | --- | --- | --- | --- | --- | --- | --- |
| Interceptor | 0.12 | | | 0.05 | | 0.28 | | -4.905 | | <0.001 | |
| Early Evening (5pm to 9pm) | 1.03 | | | 0.41 | | 2.60 | | 0.072 | | 0.943 | |
| Late Evening (9pm to 12am) | 1.21 | | | 0.48 | | 3.06 | | 0.400 | | 0.689 | |
| Middle of Night (12am to 3am) | 0.90 | | | 0.30 | | 2.68 | | -0.190 | | 0.849 | |
| Early Morning (3am to 7am) | Ref. | | | Ref. | | Ref. | |  | |  | |

**Supplementary Table 13b.** Full logistic regression model of sugar feeding by *An. gambiae* females based on time of collection where the outcome is any sugar feeding (anthrone result = 1, 2, or 3) versus no sugar feeding (anthrone result = 0).

| **Collection Time** | **Collection Time**  **Estimate** | **Reference** | **Reference**  **Estimate** | **Odds Ratio** | **LowerCL** | **UpperCL** | **χ^2^** | **P-Value** |
| --- | --- | --- | --- | --- | --- | --- | --- | --- |
| Early Evening  (5pm to 9pm) | 10.4 (6.8-14) | Early Morning  (3am to 7am) | 8.2 (1-15.4) | 1.034 | 0.412 | 2.6 | 0.005 | 0.943 |
| Early Evening  (5pm to 9pm) | 10.4 (6.8-14) | Late Evening  (9pm to 12am) | 16.3 (10.1-22.5) | 0.856 | 0.505 | 1.45 | 0.335 | 0.563 |
| Early Evening  (5pm to 9pm) | 10.4 (6.8-14) | Middle of Night  (12am to 3am) | 9.6 (4.5-14.8) | 1.15 | 0.527 | 2.509 | 0.123 | 0.726 |
| Late Evening  (9pm to 12am) | 16.3 (10.1-22.5) | Early Morning  (3am to 7am) | 8.2 (1-15.4) | 1.208 | 0.478 | 3.056 | 0.160 | 0.689 |
| Late Evening  (9pm to 12am) | 16.3 (10.1-22.5) | Middle of Night  (12am to 3am) | 9.6 (4.5-14.8) | 1.343 | 0.611 | 2.952 | 0.540 | 0.462 |
| Middle of Night  (12am to 3am) | 9.6 (4.5-14.8) | Early Morning  (3am to 7am) | 8.2 (1-15.4) | 0.9 | 0.302 | 2.677 | 0.036 | 0.849 |

**Supplementary Table 14a.** Full logistic regression model of sugar feeding by *An. gambiae* males based on time of collection where the outcome is any sugar feeding (anthrone result = 1, 2, or 3) versus no sugar feeding (anthrone result = 0).

| **Parameter** | | **Odds Ratio** | **LowerCL** | | **UpperCL** | | **Z** | | **P-Value** | |  |
| --- | --- | --- | --- | --- | --- | --- | --- | --- | --- | --- | --- |
| Interceptor | 0.15 | | | 0.04 | | 0.50 | | -3.061 | | 0.002 | |
| Early Evening (5pm to 9pm) | 1.86 | | | 0.49 | | 7.09 | | 0.909 | | 0.363 | |
| Late Evening (9pm to 12am) | 0.68 | | | 0.12 | | 3.71 | | -0.450 | | 0.653 | |
| Middle of Night (12am to 3am) | 1.04 | | | 0.17 | | 6.54 | | 0.044 | | 0.965 | |
| Early Morning (3am to 7am) | Ref. | | | Ref. | | Ref. | |  | |  | |

**Supplementary Table 14b.** Full logistic regression model of sugar feeding by *An. gambiae* males based on time of collection where the outcome is any sugar feeding (anthrone result = 1, 2, or 3) versus no sugar feeding (anthrone result = 0).

| **Collection Time** | **Collection Time**  **Estimate** | **Reference** | **Reference**  **Estimate** | **Odds Ratio** | **LowerCL** | **UpperCL** | **χ^2^** | **P-Value** |
| --- | --- | --- | --- | --- | --- | --- | --- | --- |
| Early Evening  (5pm to 9pm) | 22.2 (11.7-32.8) | Early Morning (3am to 7am) | 13 (0-27.3) | 1.86 | 0.488 | 7.094 | 0.826 | 0.363 |
| Early Evening  (5pm to 9pm) | 22.2 (11.7-32.8) | Late Evening (9pm to 12am) | 9.1 (0-19) | 2.749 | 0.762 | 9.912 | 2.388 | 0.122 |
| Early Evening  (5pm to 9pm) | 22.2 (11.7-32.8) | Middle of Night (12am to 3am) | 15 (0-31.9) | 1.785 | 0.416 | 7.661 | 0.607 | 0.436 |
| Late Evening  (9pm to 12am) | 9.1 (0-19) | Early Morning (3am to 7am) | 13 (0-27.3) | 0.677 | 0.123 | 3.709 | 0.202 | 0.653 |
| Late Evening  (9pm to 12am) | 9.1 (0-19) | Middle of Night (12am to 3am) | 15 (0-31.9) | 0.649 | 0.108 | 3.911 | 0.222 | 0.637 |
| Middle of Night  (12am to 3am) | 15 (0-31.9) | Early Morning (3am to 7am) | 13 (0-27.3) | 1.042 | 0.166 | 6.538 | 0.002 | 0.965 |

**Supplementary Table 15a.** Full logistic regression model of sugar feeding by *An. coustani* females based on time of collection where the outcome is any sugar feeding (anthrone result = 1, 2, or 3) versus no sugar feeding (anthrone result = 0).

| **Parameter** | | **Odds Ratio** | **LowerCL** | | **UpperCL** | | **Z** | | **P-Value** | |  |
| --- | --- | --- | --- | --- | --- | --- | --- | --- | --- | --- | --- |
| Interceptor | 0.15 | | | 0.12 | | 0.19 | | -16.859 | | <0.001 | |
| Early Evening (5pm to 9pm) | 0.67 | | | 0.49 | | 0.90 | | -2.606 | | 0.009 | |
| Late Evening (9pm to 12am) | 0.48 | | | 0.36 | | 0.64 | | -5.07 | | <0.001 | |
| Middle of Night (12am to 3am) | 0.57 | | | 0.41 | | 0.81 | | -3.156 | | 0.002 | |
| Early Morning (3am to 7am) | Ref. | | | Ref. | | Ref. | |  | |  | |

**Supplementary Table 15b.**  Full logistic regression model of sugar feeding by *An. coustani* females based on time of collection where the outcome is any sugar feeding (anthrone result = 1, 2, or 3) versus no sugar feeding (anthrone result = 0).

| **Collection Time** | **Collection Time**  **Estimate** | **Reference** | **Reference**  **Estimate** | **Odds Ratio** | **LowerCL** | **UpperCL** | **χ^2^** | **P-Value** |
| --- | --- | --- | --- | --- | --- | --- | --- | --- |
| Early Evening  (5pm to 9pm) | 8.4*(6.6-10.1) | Early Morning (3am to 7am) | 12.8*(10.4-15.3) | 0.667 | 0.492 | 0.904 | 6.794 | **0.009** |
| Early Evening  (5pm to 9pm) | 8.4*(6.6-10.1) | Late Evening (9pm to 12am) | 7.2*(5.9-8.4) | 1.396 | 1.053 | 1.849 | 5.392 | 0.020 |
| Early Evening  (5pm to 9pm) | 8.4*(6.6-10.1) | Middle of Night (12am to 3am) | 7.9*(5.9-10) | 1.16 | 0.825 | 1.631 | 0.732 | 0.392 |
| Late Evening  (9pm to 12am) | 7.2*(5.9-8.4) | Early Morning (3am to 7am) | 12.8*(10.4-15.3) | 0.478 | 0.359 | 0.636 | 25.706 | **<0.001** |
| Late Evening  (9pm to 12am) | 7.2*(5.9-8.4) | Middle of Night (12am to 3am) | 7.9*(5.9-10) | 0.831 | 0.602 | 1.149 | 1.249 | 0.264 |
| Middle of Night  (12am to 3am) | 7.9*(5.9-10) | Early Morning (3am to 7am) | 12.8*(10.4-15.3) | 0.575 | 0.408 | 0.811 | 9.963 | **0.002** |

**Supplementary Table 16a.** Full logistic regression model of sugar feeding by *An. coustani* males based on time of collection where the outcome is any sugar feeding (anthrone result = 1, 2, or 3) versus no sugar feeding (anthrone result = 0).

| **Parameter** | | **Odds Ratio** | **LowerCL** | | **UpperCL** | | **Z** | | **P-Value** | |  |
| --- | --- | --- | --- | --- | --- | --- | --- | --- | --- | --- | --- |
| Interceptor | 0.19 | | | 0.12 | | 0.31 | | -7.098 | | <0.001 | |
| Early Evening (5pm to 9pm) | 0.47 | | | 0.27 | | 0.84 | | -2.57 | | 0.010 | |
| Late Evening (9pm to 12am) | 0.53 | | | 0.29 | | 0.96 | | -2.102 | | 0.036 | |
| Middle of Night (12am to 3am) | 0.66 | | | 0.36 | | 1.20 | | -1.372 | | 0.170 | |
| Early Morning (3am to 7am) | Ref. | | | Ref. | | Ref. | |  | |  | |

**Supplementary Table 16b.** Full logistic regression model of sugar feeding by *An. coustani* males based on time of collection where the outcome is any sugar feeding (anthrone result = 1, 2, or 3) versus no sugar feeding (anthrone result = 0).

| **Collection Time** | **Collection Time**  **Estimate** | **Reference** | **Reference**  **Estimate** | **Odds Ratio** | **LowerCL** | **UpperCL** | **χ^2^** | **P-Value** |
| --- | --- | --- | --- | --- | --- | --- | --- | --- |
| Early Evening  (5pm to 9pm) | 7.6*(5-10.2) | Early Morning (3am to 7am) | 14.9*(8.7-21.2) | 0.473 | 0.267 | 0.837 | 6.607 | **0.010** |
| Early Evening  (5pm to 9pm) | 7.6*(5-10.2) | Late Evening (9pm to 12am) | 9.3*(6-12.7) | 0.89 | 0.533 | 1.486 | 0.199 | 0.656 |
| Early Evening  (5pm to 9pm) | 7.6*(5-10.2) | Middle of Night (12am to 3am) | 10.5*(6.9-14.2) | 0.717 | 0.427 | 1.204 | 1.585 | 0.208 |
| Late Evening  (9pm to 12am) | 9.3*(6-12.7) | Early Morning (3am to 7am) | 14.9*(8.7-21.2) | 0.532 | 0.295 | 0.958 | 4.419 | **0.036** |
| Late Evening  (9pm to 12am) | 9.3*(6-12.7) | Middle of Night (12am to 3am) | 10.5*(6.9-14.2) | 0.806 | 0.47 | 1.38 | 0.62 | 0.431 |
| Middle of Night  (12am to 3am) | 10.5*(6.9-14.2) | Early Morning (3am to 7am) | 14.9*(8.7-21.2) | 0.66 | 0.364 | 1.195 | 1.881 | 0.170 |
